# Alu hypomethylation and MGMT hypermethylation in serum as biomarkers of glioma

**DOI:** 10.18632/oncotarget.20012

**Published:** 2017-08-07

**Authors:** Mingjie Gong, Wei Shi, Jing Qi, Guoping Shao, Zhenghua Shi, Junxiang Wang, Jian Chen, Rongtao Chu

**Affiliations:** ^1^ Department of Neurosurgery, Changshu No. 2 People's Hospital (The 5th Clinical Medical College of Yangzhou University), Changshu, Jiangsu Province, China; ^2^ Department of Neurosurgery, Affiliated Hospital of Nantong University, Nantong, Jiangsu Province, China; ^3^ Comprehensive Surgical Laboratory, Affiliated Hospital of Nantong University, Nantong, Jiangsu Province, China

**Keywords:** Alu, MGMT, serum, DNA, glioma

## Abstract

In order to improve prognosis of glioma patients, better tools are required for early diagnosis and treatment. Serum cell-free DNA methylation levels of Alu, MGMT, P16, RASSF1A from 124 glioma patients and 58 healthy controls were detected by the bisulfite sequencing. The median methylation level of Alu was 46.15% (IQR, 36.57%–54.00%) and 60.85% (IQR, 57.23%–65.68%) in glioma patients and healthy controls respectively. The median methylation level of MGMT in glioma samples was 64.65% (IQR, 54.87%–74.37%) compared to 38.30% (IQR, 34.13%–45.45%) in healthy controls, and all revealed significant differences including P16. However, the median methylation level of RASSF1A was not significantly altered in glioma patients. Furthermore, the methylation levels of Alu and MGMT in serum had a good diagnostic value, and was higher than P16. Interestingly, combination of Alu and MGMT identified additional patients, which were missed by either diagnosis alone. In the Alu group, the patients with high levels were associated with an increased survival rate compared to those who with low levels, with similar results observed in the MGMT group. In the present study, we demonstrated that the methylation level of Alu and MGMT in serum had a better diagnostic value than P16. Moreover, combined analysis of Alu and MGMT showed higher sensitivity for glioma diagnosis. Therefore, both serum Alu and MGMT methylation levels may represent a novel prognostic factor for glioma patients.

## INTRODUCTION

Glioma is the most common primary brain tumor, and is associated with a poor prognosis in part due to of its invasive nature. Early diagnosis and prompt treatment of glioma can be used to reduce mortality and improve prognosis. However, at present, the diagnosis of glioma mainly depends on imaging examination, which may misrepresent true intracranial tumor burden and phenotype. Although biopsy is used to confirm diagnosis of glioma, it is an invasive approach. Tumor biomarkers such as Alpha Fetoprotein, Carcino Embryonie Antigen, Cancer Antigen 125 have been reported to be useful to screen early malignancy and aid in cancer diagnosis without invasiveness. Furthermore, following a diagnosis of cancer, biomarkers may be used in order to determine prognosis and predict therapeutic response [1, 2]. Unfortunately, to date, there is no such biomarker for glioma patients. Therefore, it is desirable to identify biomarkers for use in the early diagnosis of glioma.

Cell-free DNA can be detected in body fluids such as serum/plasma, urine and sputum, and are released into the blood following apoptosis and necrosis of cancer cells in the tumor microenvironment. Therefore, there are greater amounts cell-free DNA in cancer patients compared to healthy controls. As is known, aberrant DNA methylation including global hypomethylation and regional hypermethylation, play an important role in the formation and progression of cancer. Over the past decades, many researchers have demonstrated the presence of aberrant methylation in the serum cell-free DNA of patients with various types of tumors [3–7]. However, few studies have investigated serum cell-free DNA in glioma. Furthermore, the published data on tumor suppressor genes *MGMT*, *RASSF1A* and *P16* are inconsistent due to the different genes or different methods [8–15]. Moreover, almost all previous studies regarding aberrant methylation in glioma are focused on promoter hypermethylation, which is generally associated with gene silencing[16–20], with is limited information on global hypomethylation in serum cell-free DNA of glioma patients to date. Global hypomethylation mainly occurs in repetitive elements and leads to genomic instability and tumorigenesis [21, 22]. As such, global hypomethylation is considered a biomarker in many tumors [23]. Alu elements are the most abundant repetitive elements in the human genome, with the estimation of total methylation content of Alu elements reported to be useful in evaluating the global genomic methylation levels. Cadieux et al [24] reported that the degree of genomic hypomethylation was related with disease stage in glioma and thus may serve as a potential prognostic factor.

In the present study, we use the bisulfite sequencing (BSP), to detect the methylation status of *Alu*, *MGMT*, *RASSF1A*, and *P16* in serum cell-free DNA of glioma patients compared to healthy controls. Identification of novel serum-based biomarkers may contribute significantly to the early diagnosis and thus improved prognosis of glioma patients.

## RESULTS

### Methylation levels of *Alu*, *MGMT*, *RASSF1A*, *P16* in serum

**Figure 1:**
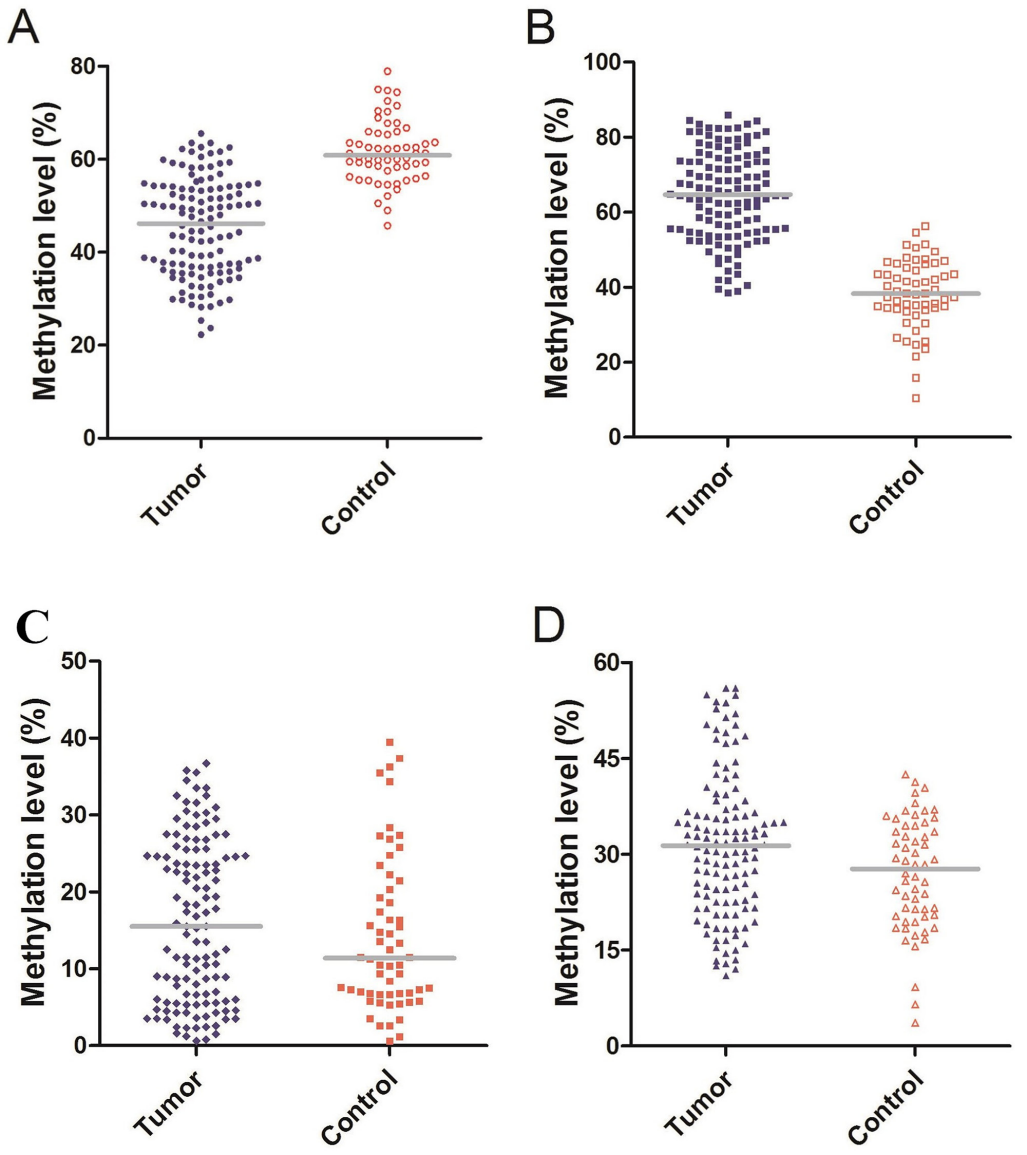
**(A)** Methylation level of *Alu*: The median methylation level of *Alu* was 46.15% (IQR, 36.57%–54.00%) and 60.85% (IQR, 57.23%–65.68%) in glioma patients and healthy controls. **(B)** Methylation level of *MGMT*: The median methylation level of *MGMT* was 64.65% (IQR, 54.87%–74.37%) and 38.30% (IQR, 34.13%–45.45%) in glioma patients and healthy controls. **(C)** Methylation level of *RASSF1A*: The median methylation level of *RASSF1A* was 15.50% (IQR, 5.85%–24.70%) and 11.40% (IQR, 6.78%–20.60%) in glioma patients and healthy controls. **(D)** Methylation level of *P16*: The median methylation level of *P16* was 31.35% (IQR, 22.58%–36.93%) and 26.65% (IQR, 17.15%–36.05%) in glioma patients and healthy controls.

The number of methylated CpG sites were defined by dividing the number of methylated CpG sites plus unmethylated CpG sites and expressed as the final methylation level (%) of each sample in the four genes. The median methylation level of *Alu* was 46.15% (IQR, 36.57%–54.00%) and 60.85% (IQR, 57.23%–65.68%) in glioma patients and healthy controls, respectively. Indeed, there was a significant difference (P<0.01, Figure 1A). The median methylation level of *MGMT* was significantly higher in glioma patients 64.65% (IQR, 54.87%–74.37%) compared to 38.30% (IQR, 34.13%–45.45%) in healthy controls (P<0.01, Figure 1B). However, the median methylation level of *RASSF1A* in patients and healthy controls did not reveal any significant differences (P=0.349), and were 15.50% (IQR, 5.85%–24.70%), 11.40% (IQR, 6.78%–20.60%), respectively (Figure 1C). In contrast to *RASSF1A*, the difference between patients and controls in P16 methylation levels was statistically significant (P=0.03), 31.35% (IQR, 22.58%–36.93%) and 26.65% (IQR, 17.15%–36.05%, Figure 1D).

### Diagnostic performance of serum cell-free DNA

**Figure 2:**
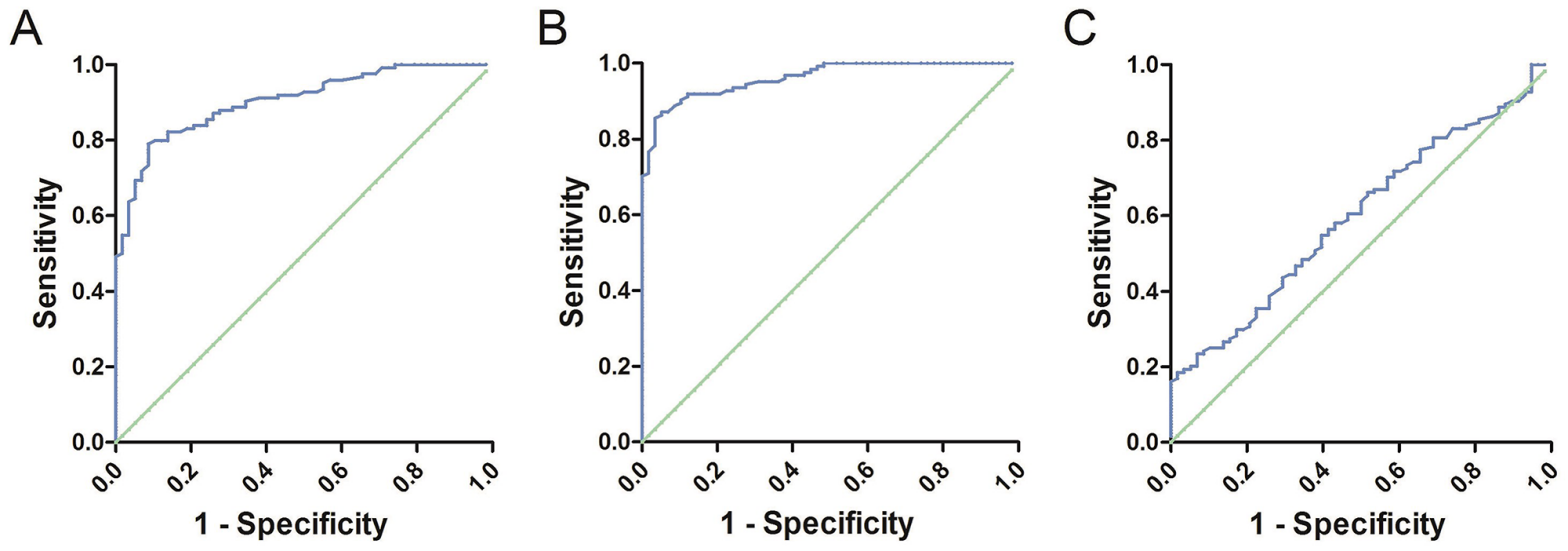
**(A)** ROC curve of *Alu*: The Area Under Curve was 0.904. **(B)** ROC curve of *MGMT*: The Area Under Curve was 0.962. **(C)** ROC curve of *P16*: The Area Under Curve was 0.599.

**Figure 3:**
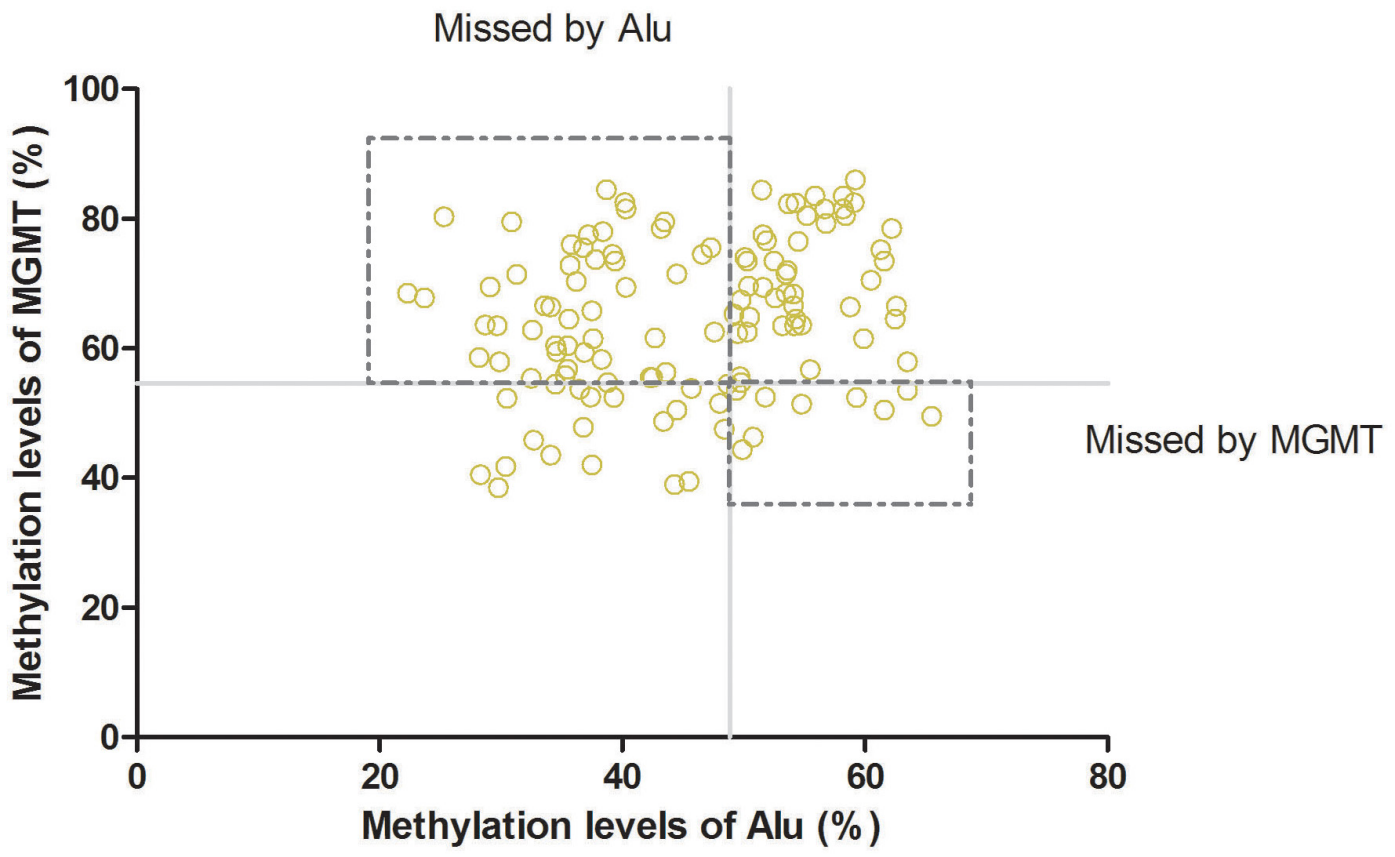
Combination of *Alu* and *MGMT:* Combination of Alu and MGMTcould improve the rate of diagnosis

ROC curves were constructed to assess the feasibility of serum cell-free DNA for the diagnosis of glioma patients. We analyzed the methylation levels of *Alu*, *MGMT*, *P16* in serum between patients and healthy controls. The Area Under Curve was 0.904 (95% CI: 0.861–0.946, P<0.01, Figure 2A) in *Alu*, 0.962 (95% CI: 0.938–0.985, P<0.01, Figure 2B) in *MGMT* and 0.599 (95% CI: 0.514–0.683, P<0.01, Figure 2C) in *P16*. These results indicated that the methylation level of *Alu* and *MGMT* in serum may have good diagnostic value that is greater than *P16*. Further analysis revealed a 54.84% sensitivity and 98.28% specificity at the optimal cutoff value of 48.85% (Youden's index) in Alu, compared to 76.61% sensitivity and 98.28% specificity at the optimal cutoff value of 48.85% (Youden's index) in *MGMT*. Consequently, combination of *Alu* and *MGMT* may identify additional patients that may be missed by either diagnosis alone (Figure 3).

### Methylation level and survival

**Figure 4:**
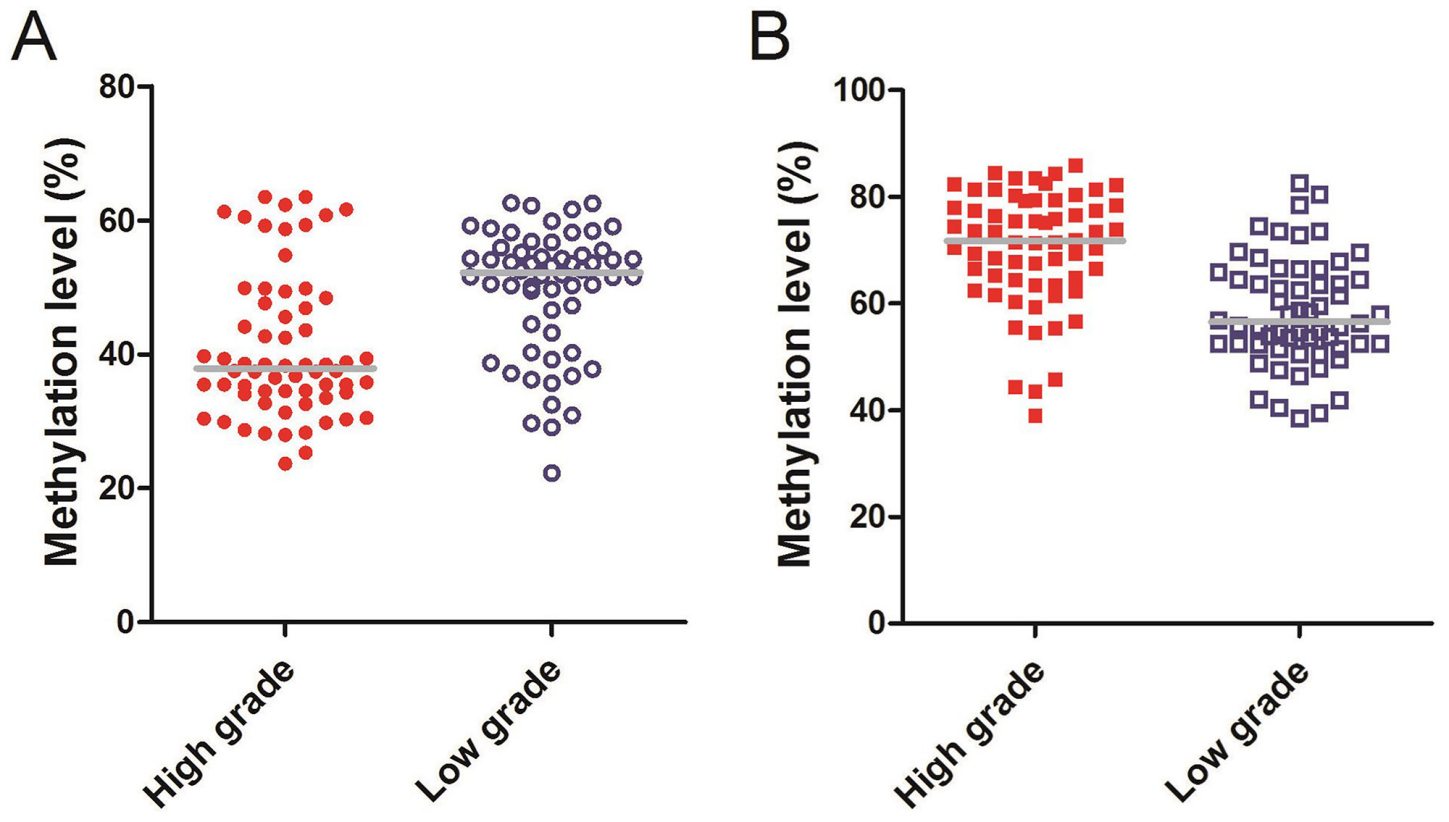
**(A)** Methylaiton level of *Alu*: The median methylation level of *Alu* in high-grade glioma was 37.90% (IQR, 34.15%–48.20%), while the low-grade was 52.20% (IQR, 43.53%–55.43%). **(B)** Methylaiton level of *MGMT*: The median methylation level of *MGMT* in high-grade glioma was 71.75% (IQR, 63.75%–75.10%), while the low-grade was 56.55% (IQR, 52.33%–65.48%).

In order to explore the relationship between serum cell-free DNA aberrant methylation and survival of glioma patients, we compared the methylation level of *Alu*, *MGMT* in different glioma grade groups and constructed the survival curves. The median methylation level of *Alu* in high-grade glioma was 37.90% (IQR, 34.15%–48.20%), while the low-grade was 52.20% (IQR, 43.53%–55.43%, P<0.01, Figure 4A). However, the median methylation level of *MGMT* in high-grade glioma was 71.75% (IQR, 63.75%–75.10%) compared to 56.55% of low-grade tumors (IQR, 52.33%–65.48%, P<0.01, Figure 4B).

**Figure 5:**
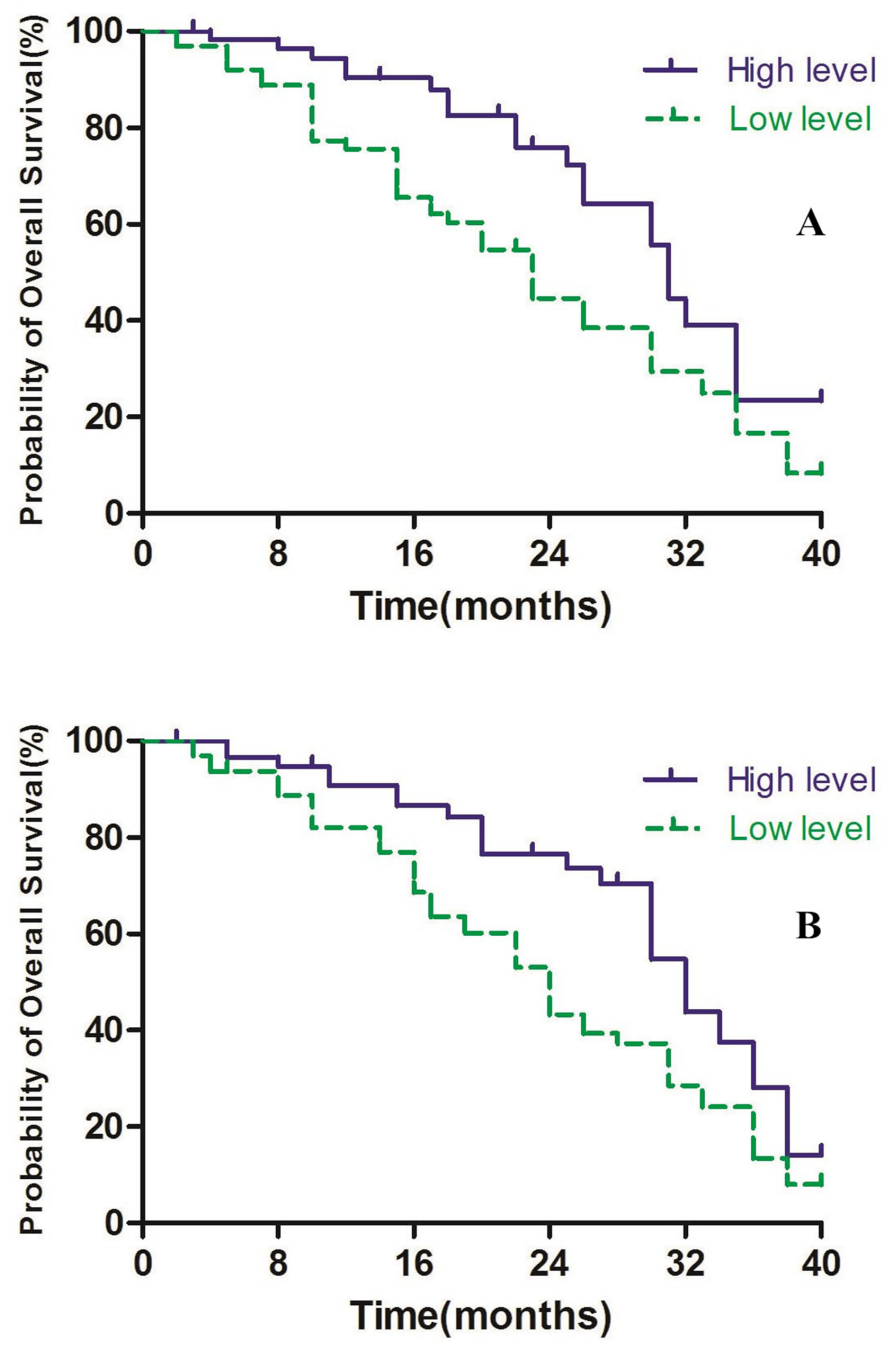
**(A)** *Alu* methylaiton level and overall survival. **(B)** *MGMT* methylaiton level and overall survival.

In the present study, we defined > 46.15% as high methylation level and ≤ 46.15% as low methylation level for *Alu*, and > 64.65% as high methylation level and ≤ 64.65% as low methylation level for *MGMT*. In Alu group, the patients with high levels were associated with higher survival rates compared to those with low levels, with median OS time was 31 months in the high level group and 23 months in low level group (P<0.01, Figure 5A). Furthermore, similar results were obtained in the *MGMT* group, with median OS time 32 months in high level group compared to 24 months in the low level group (P<0.01, Figure 5B).

## DISCUSSION

Early diagnosis and treatment plays a critical role in improving prognosis for glioma patients. However, currently there are no tumor-specific biomarkers for glioma to assist in early diagnosis. Previous studies[25–30] have reported that aberrant methylation of serum cell-free DNA offers the possibility to identify biomarkers for glioma early diagnosis and improve prognosis. Additionally, the detection of serum cell-free DNA as a noninvasive diagnostic method may provide an alternate diagnostic method to tumor tissue biopsy. Moreover, serum cell-free DNA is very stable and can survive harsh conditions for long time. In addition, serum cell-free DNA can be amplified by PCR or other technologies even if only small amounts are present. Moreover, aberrant methylation including global hypomethylation and regional hypermethylation occur in the early stages of tumorigenesis, and thus exist both in early and advanced cancers [31]. Furthermore, this study is the first to detect global hypomethylation and regional hypermethylation in serum at the same time.

In the present study, we used BSP which is the gold-standard technology to detect the methylation status of serum cell-free DNA, and it could sequence more CpG sites to get more accurate results than other technologies. We detected four genes (*Alu*, *MGMT*, *RASSF1A*, *P16*) in the serum of 124 glioma patients and 58 healthy controls. Results revealed the methylation levels of *Alu*, *MGMT* and *P16* had significant differences between patients and controls. Cadieux et al[24] reported that *Alu* hypomethylation in glioblastoma multiforme, consistent with the results of our study, which demonstrated that Alu was hypomethylation in glioma patients and heavily methylated in healthy people. Similarly, we also found *MGMT* hypermethylation in patients while hypomethylation in controls consistent with previous reports [32–34]. P16 has been reported as hypermethylation in glioma [11], with the present study demonstrating a similar trend that was not significantly different. However, this study obtained different results to the literature in *RASSF1A* [19, 35, 36], as we did not identify *RASSF1A* hypermethylation in patients and hypomethylation in healthy controls. For further analysis of diagnostic value of serum cell-free DNA for glioma, we constructed the ROC curves, the AUC of *Alu*, *MGMT*, *P16* was 0.904, 0.962, 0.599, respectively and identified that the methylation levels of *Alu* and *MGMT* in the serum had better diagnostic value than *P16*. Furthermore, combined analysis of *Alu* and *MGMT* revealed higher sensitivity for glioma diagnosis.

To evaluate the prognostic significance of *Alu* and *MGMT*, we analyzed the methylation level of *Alu* and *MGMT* in high-grade and low-grade groups. In Alu, the low-grade glioma patients had higher levels than high-grade group. The high-grade group had higher level than low-grade group in *MGMT*. Moreover, we performed the overall survival curves according to the follow-up data of patients. In the *Alu* group, results showed that patients in the high level group had higher survival rates compared to the low level group. *MGMT* analysis, also showed that the high level group had longer survival time compared to the low level group, perhaps due to epigenetically silenced *MGMT* treated with radiotherapy and alkylating chemotherapy may improve the survival of patients with glioblastoma without *MGMT* expression [37, 38].

In the present study, we detected increased global hypomethylation (*Alu*) and regional hypermethylation (*MGMT*, *RASSF1A*, *P16*) in serum cell-free DNA of glioma patients compared to healthy controls, and demonstrate that both *Alu* and *MGMT* may have good diagnostic value. However, our results suggest that there was much better diagnostic efficiency if a combination of *Alu* and *MGMT* was used. Therefore, both serum *Alu* and *MGMT* methylation level could be useful tools to predict the prognosis of glioma patients. But could patients that have hypermethylated MGMT with hypomethylated Alu have worst overall survival than patents with only hypermethylated MGMT, hypomethylated Alu only, and hypomethylated MGMT with hypermethylated Alu? We plan to increase the number of patients in the future research for getting more real and effective results.

## MATERIALS AND METHODS

### Patients

The study involved 124 glioma patients, treated at Department of Neurosurgery at the Changshu No.2 People's Hospital/ The 5^th^ Affiliated Hospital of Yangzhou University (Changshu, People's Republic of China) and the Affiliated Hospital of Nantong University (Nantong, People's Republic of China) between 2003 and 2012. The sample population contained 60 low-grade gliomas (32 grade I, 28 grade II), and 64 high-grade gliomas (34 grade III, 30 grade IV). All patients included in the study underwent surgical tumor resection, and diagnosis of glioma was confirmed by histological examination. Patients then underwent the accepted standard radiotherapy and chemotherapy if necessary. The following clinical data determined for patients are presented in Table 1: age at the time of operation, sex, Karnofsky performance score, tumor diameter, tumor location (region, side) and the World Health Organization grade. The study protocol was approved by the Independent Ethics Committee of Affiliated Hospital of Nantong University, with all patients and healthy controls providing informed consent prior to obtaining the serum samples.

**Table 1:** Patients’ chracteristics

Characteristic	No. of patients (%)
Age at diagnosis, years	
≤50	46 (37.10)
<50	78 (62.90)
Gender	
Male	55 (44.35)
Female	69 (55.65)
Karnofsky performance score(KPS)	
<70	26 (20.97)
≥70	98 (79.03)
Tumor diameter	
≤3cm	39 (31.45)
<3cm	85 (68.55)
Tumor location (region)	
Frontal	45 (36.29)
Temporal	37 (29.84)
Parietal	29 (23.39)
Occipital	10 (8.06)
Other	3 (2.42)
Tumor location (side)	
Left	38 (30.65)
Right	46 (37.10)
Middle	40 (32.26)
WHO grade	
Low grade	60 (48.39)
High grade	64 (51.61)

### Cell-free DNA extraction and sodium bisulfite modification

Peripheral venous blood (5–10 ml) was collected prior to surgery from 124 glioma patients and 58 blood samples from healthy people were also obtained as controls. Serum was isolated by centrifugation at 3500 rpm for 10 min at room temperature and stored at −80°C until use. Serum cell-free DNA was obtained by QIAamp MinElute Virus Spin Kit (Qiagen, Germany), according to the manufacturer's instructions and stored at −20°C until use. Sodium bisulfite modification of cell-free DNA was performed with Epitect Bisulfite Kit (Qiagen) according to the manufacturer's instructions. Briefly, 1 μg prepared DNA were bisulfite treated to convert unmethylated cytosine to uracil and the elutions were stored at −20°C until use.

### PCR of Alu, MGMT, RASSF1A and P16

The primers of *Alu*, *MGMT*, *RASSF1A* and *P16* for BSP were designed using Methyl Primer Express® Software v1.0. Amplification of the four genes used a 50 μl PCR mixture, which contained 5 μl Buffer, 4 μl dNTP, 0.5 μl HotStar Taq DNA polymerase (Invitrogen, USA), 0.8 μl (10 μM) forward and reverse primers, 10 μl bisulfite converted DNA and 28.9 μl ddH_2_O. The PCR of Alu was performed as follows: initial denaturing at 94°C for 3 min; 37 cycles of denaturing at 94°C for 20 s, annealing at 55°C for 30 s, extension for 30 s at 72°C, and a final extension at 72°C for 5 min. The PCR cycling conditions of RASSF1A were 94°C for 2 min, 37 cycles at 94°C for 20 s, 57°C for 30 s, and 72°C for 30 s, followed by 72°C for 10 min. The PCR products were analyzed on a 1.5% agarose gel stained with ethidium bromide and subjected to UV illumination. (*MGMT*, *P16*)

### Cloning and sequencing

The separated PCR products by the gel were purified using QIAquick Gel Extraction kit (Qiagen) and then cloned using the TOPO TA-cloning kit (Invitrogen) according to the manufacturer's instructions. Colony PCR was undertaken to screen the positive colonies, and the clones of the right size of PCR product were sequenced using the ABI 3730 DNA Analyzer. Additionally, 10 clones of each sample were subjected to standard sequencing analysis.

### Statistical analysis

Analysis of all data was performed with GraphPad Prism software (version 5.01). Comparison between two groups was tested by using the t test or Mann–Whitney test. Receiver Operator Characteristic curve curve was constructed to assess the diagnostic value of serum cell-free DNA for glioma. Overall survival (OS) was calculated from the day of first surgery until death or end of follow-up, and analysis performed by Kaplan–Meier and the log rank test. The level of statistical significance was set at P<0.05.

## Abbreviations

MGMT: O6-methylguanine-DNA-methyltransferase
RASSFIA: ras association domain family gene 1A
IQR: interquartile range
BSP: bisulfite sequencing
ROC: receiver operator characteristic
AUC: area under curve
OS: overall survival
KPS: Karnofsky performance score
